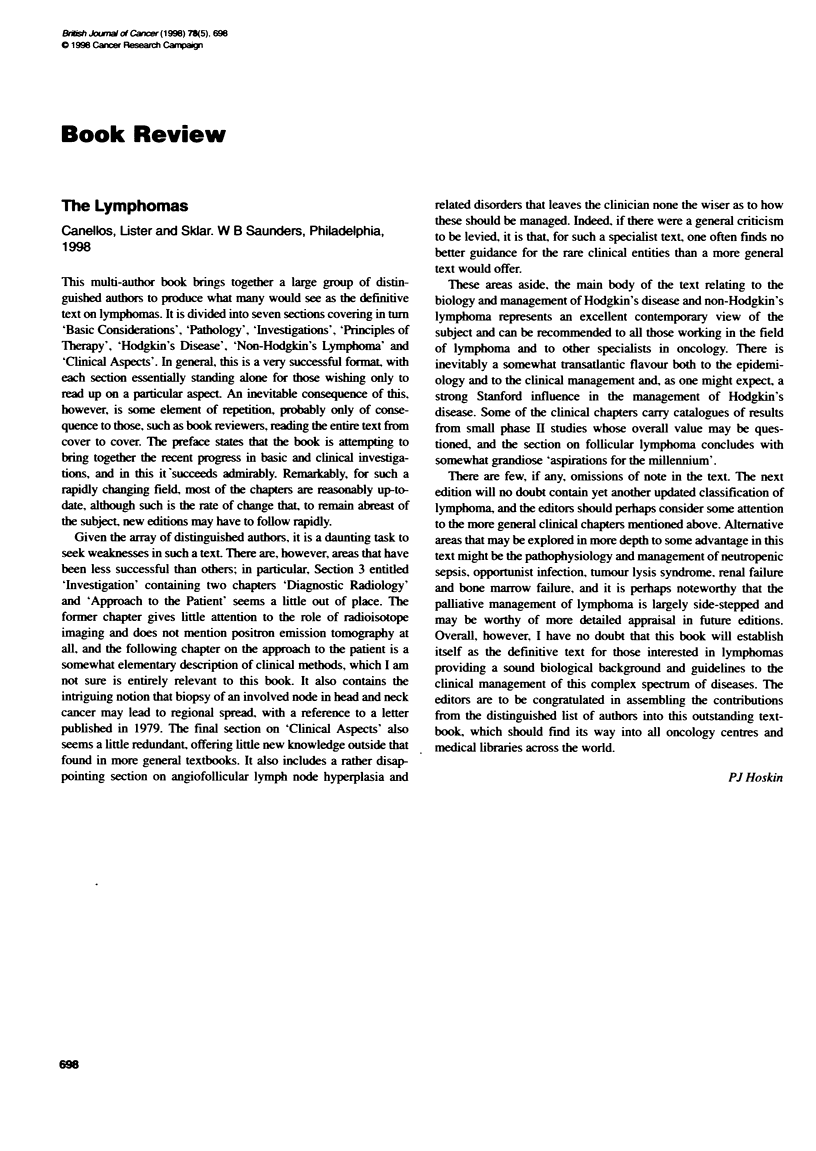# The Lymphomas

**Published:** 1998-09

**Authors:** P J Hoskin


British Journal of Cancer (1998) 78(5), 698

© 1998 Cancer Research Campaign

### Book Review

### The Lymphomas

Canellos, Lister and Sklar. W B Saunders, Philadelphia, 1998

This multi-author book brings together a large group of distinguished authors to produce what many would see as the definitive text on lymphomas. It is divided into seven sections covering in turn 'Basic Considerations', 'Pathology', 'Investigations', 'Principles of Therapy', 'Hodgkin's Disease', 'Non-Hodgkin's Lymphoma' and 'Clinical Aspects'. In general, this is a very successful format, with each section essentially standing alone for those wishing only to read up on a particular aspect. An inevitable consequence of this, however, is some element of repetition, probably only of consequence to those, such as book reviewers, reading the entire text from cover to cover. The preface states that the book is attempting to bring together the recent progress in basic and clinical investigations, and in this it succeeds admirably. Remarkably, for such a rapidly changing field, most of the chapters are reasonably up-to-date, although such is the rate of change that, to remain abreast of the subject, new editions may have to follow rapidly.

Given the array of distinguished authors, it is a daunting task to seek weaknesses in such a text. There are, however, areas that have been less successful than others; in particular, Section 3 entitled 'Investigation' containing two chapters 'Diagnostic Radiology' and 'Approach to the Patient' seems a little out of place. The former chapter gives little attention to the role of radioisotope imaging and does not mention positron emission tomography at all, and the following chapter on the approach to the patient is a somewhat elementary description of clinical methods, which I am not sure is entirely relevant to this book. It also contains the intriguing notion that biopsy of an involved node in head and neck cancer may lead to regional spread, with a reference to a letter published in 1979. The final section on 'Clinical Aspects' also seems a little redundant, offering little new knowledge outside that found in more general textbooks. It also includes a rather disappointing section on angiofollicular lymph node hyperplasia and related disorders that leaves the clinician none the wiser as to how these should be managed. Indeed, if there were a general criticism to be levied, it is that, for such a specialist text, one often finds no better guidance for the rare clinical entities than a more general text would offer.

These areas aside, the main body of the text relating to the biology and management of Hodgkin's disease and non-Hodgkin's lymphoma represents an excellent contemporary view of the subject and can be recommended to all those working in the field of lymphoma and to other specialists in oncology. There is inevitably a somewhat transatlantic flavour both to the epidemiology and to the clinical management and, as one might expect, a strong Stanford influence in the management of Hodgkin's disease. Some of the clinical chapters carry catalogues of results from small phase II studies whose overall value may be questioned, and the section on follicular lymphoma concludes with somewhat grandiose 'aspirations for the millennium'.

There are few, if any, omissions of note in the text. The next edition will no doubt contain yet another updated classification of lymphoma, and the editors should perhaps consider some attention to the more general clinical chapters mentioned above. Alternative areas that may be explored in more depth to some advantage in this text might be the pathophysiology and management of neutropenic sepsis, opportunist infection, tumour lysis syndrome, renal failure and bone marrow failure, and it is perhaps noteworthy that the palliative management of lymphoma is largely side-stepped and may be worthy of more detailed appraisal in future editions. Overall, however, I have no doubt that this book will establish itself as the definitive text for those interested in lymphomas providing a sound biological background and guidelines to the clinical management of this complex spectrum of diseases. The editors are to be congratulated in assembling the contributions from the distinguished list of authors into this outstanding textbook, which should find its way into all oncology centres and medical libraries across the world.

*PJ Hoskin*